# Neural Injuries Induced by Hydrostatic Pressure Associated With Mass Effect after Intracerebral Hemorrhage

**DOI:** 10.1038/s41598-018-27275-7

**Published:** 2018-06-15

**Authors:** Tingwang Guo, Peng Ren, Xiaofei Li, Tiantian Luo, Yuhua Gong, Shilei Hao, Bochu Wang

**Affiliations:** 1 Key Laboratory of Biorheological Science and Technology, Ministry of Education, College of Bioengineering, Chongqing University, Chongqing, 400030 China; 2 Collaborative Innovation Center for Brain Science, Chongqing University, Chongqing, 400030 China

## Abstract

Mass effect induced by growing hematoma is one of the mechanisms by which intracerebral hemorrhage (ICH) may result in brain injuries. Our goal was to investigate the damage mechanism of hydrostatic pressure associated with mass effect and the cooperative effect of hydrostatic pressure plus hemoglobin on neural injuries. Loading hydrostatic pressure on neurons and injecting agarose gel in the right striatum of rats was performed to establish the *in vitro* and *vivo* ICH models, respectively. The elevated hydrostatic pressure associated with ICH suppressed neurons and neural tissues viability, and disturbed the axons and dendrites *in vitro* and *vivo*. Moreover, hydrostatic pressure could upregulate the expression of cleaved-caspase-3 and BAX, and downregulate Bcl-2 and Bcl-xL. Meanwhile, the toxicity of hemoglobin would be enhanced when conducted with hydrostatic pressure together. Furthermore, the exclusive hydrostatic pressure could upregulate the Piezo-2 expression, which reached a plateau at 8 h after ICH. And hemoglobin increased Piezo-2 expression significantly *in vivo*, and that was also promoted significantly by the elevated volume of Gel in the cooperative groups. Results indicated that hydrostatic pressure induced by mass effect not only gave rise to brain injuries directly, but also increased the toxicity of hemoglobin in the progress of secondary brain injury after ICH.

## Introduction

Intracerebral hemorrhage (ICH), a subtype of stroke, is associated with high mortality and disability^1^, and no widely approved and valid therapeutic managements are available to improve the severe neurological deficits and bad outcomes after ICH^2^. Understanding mechanisms underlying brain injury induced by ICH have the potential to provide the new targets and develop the effective treatments. Most of studies have focued on the effect of coagulation cascade (especially thrombin), hemoglobin breakdown products, and inflammation on the brain injuries after ICH^3,4^. However, it is generally believed that mass effect resulting from hematoma is also one of the mechanisms by which ICH may induce brain injuries within 4 h from the onset, and large volume hematoma is commonly associated with high intracranial pressure (ICP), brain tissue shifts and bad outcomes^5,6^. Nevertheless, the mechanism of neural injuries induced by mass effect after ICH is not clear.

Mass effect from hematoma and physical disruption from adjacent tissues can be defined as the primary brain injury^7,8^. In addition, brain edema would also develop immediately after the onset of ICH^8^. The direct mass effects and edema formation following ICH increase ICP and exert mechanical effects on neural network^6^. The elevated ICP would result in the increased hydrostatic pressure, and affect the ambient pressure of neurons^9^. Moreover, the differential hydrostatic pressure gradients can be generated due to the growing volume of hematoma and edema^10,11^. Researches showed that the hydrostatic pressure could even as high as 90 kPa in brain^12^. ICP greater than 20 mmHg (about 2.7 kPa) was significantly related to the mortality and poor outcomes^13,14^. In researches, the hydrostatic pressure can be simulated by the custom-designed device *in vitro* and ICH animal model *in vi*vo^15–17^. Results showed that the cell viability, mitochondrial and membranal dysfunction were influenced by hydrostatic pressure in the range of kPa to MPa for transient or long time. Besides, constant or fluctuating hydrostatic pressure has also been used to simulate the elevated intraocular pressure in glaucoma^18^, and the results suggested that hydrostatic pressure could result in severe neurologic injuries *in vitro* and *vivo* independently.

ICH not only causes primary brain injury through the direct mechanical effects of the hematoma, but also leads to secondary brain injury which are insulted by products of coagulation and hemoglobin breakdown, in particular thrombin^8,19^. So the basic research and clinical management focus on products from blood, cascades of clot components and the related inflammation^20–22^. Thrombin is an essential cascade of clot component and has an important role in the neurological injuries^23,24^. It triggers the apoptosis of neural cells, disturbs the blood brain barrier, initiates the early brain edema, and contributes to hydrocephalus through thrombin receptor. Hemoglobin^7,25,26^, as a product of blood, induces a dose- and time-dependent cytotoxicity to cortical neurons and triggers the delayed brain edema^3^. Hemoglobin can degrade into heme and iron which have detrimental effect in secondary injury. The mass effect after ICH would continue for days until the hematoma was removed by surgery, however, few researches focused on the hydrostatic pressure and its cooperative effects with hemoglobin on the neural injury.

Therefore, the *in vitro* and *vivo* models were established in this study to investigate the effect of hydrostatic pressure after ICH on primary cortical neurons or neural tissues, and we also studied whether hydrostatic pressure and hemoglobin had effect in an independent or cooperative manner. The neural viability, cellular morphology or tissue architecture and apoptosis or necrosis were detected. Further, Piezo-mediated mechanotransduction was also investigated.

## Results

### Neurons Viability *in Vitro* and Neural Activity *in Vivo*

**Figure 1:**
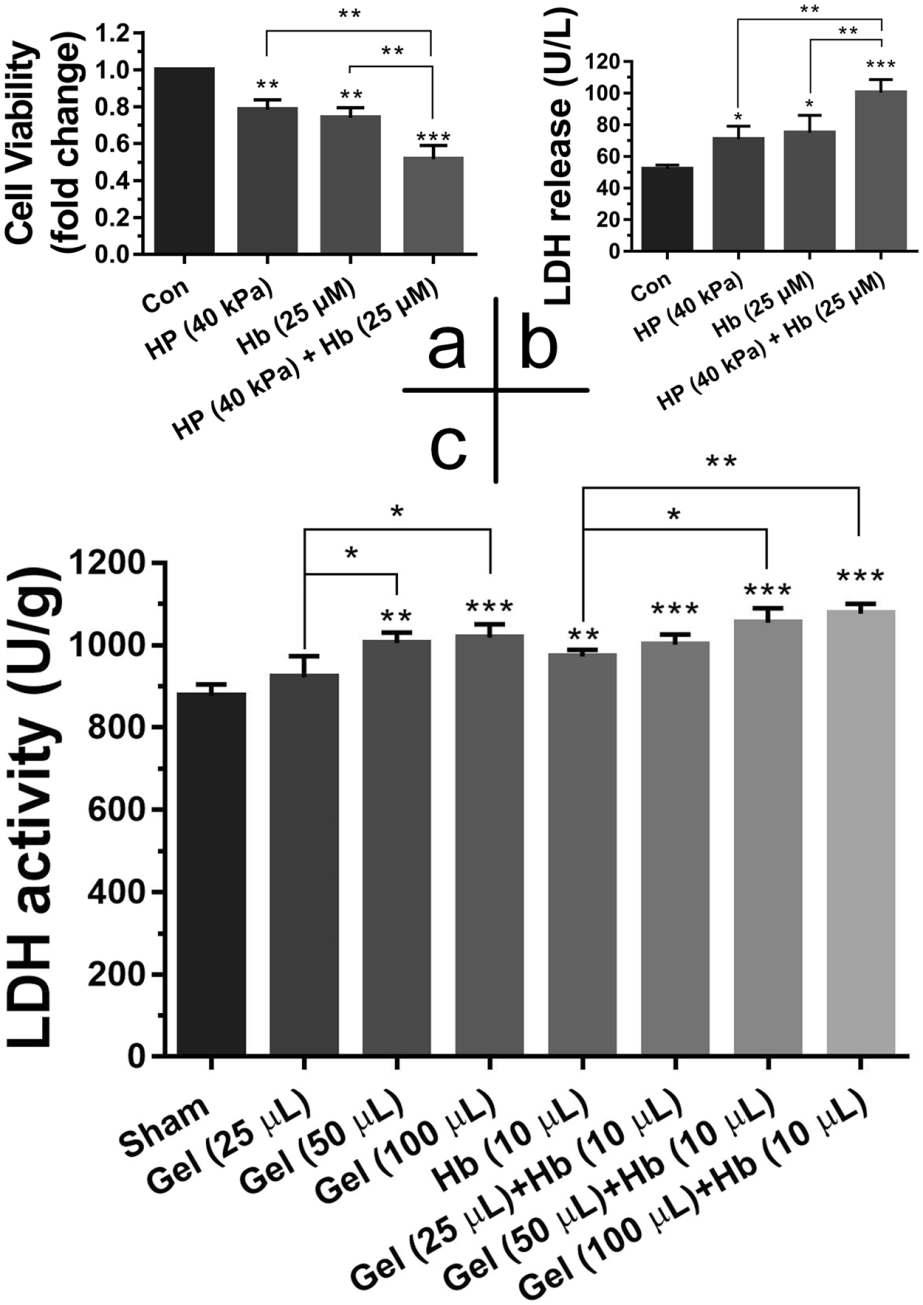
Effect of hydrostatic pressure and hemoglobin on neural viability and cytoplasmic LDH release *in vitro* (**a**,**b**) and *in vivo* (**c**). Data are expressed as the means ± SD (n = 6) (*P < 0.05, **P < 0.01, and ***P < 0.001).

The custom-designed pressure device was used to load hydrostatic pressure on neurons *in vitro*. Compared to the control group, a significant decrease in the neurons viability was observed when neurons treated with high hydrostatic pressure (40 kPa) for 24 h (see Supplementary Fig. S1a and e). In addition, hydrostatic pressure of 40 kPa led to the increase of lactate dehydrogenase (LDH) releasing (approximately 1.4-fold, see Supplementary Fig. S1b and f), which can be used to assess the level of neurological injuries. Meanwhile, the neuron viability significantly decreased in a dose-dependent manner after incubation with hemoglobin for 24 h (see Supplementary Fig. S1c), and the release of LDH significantly increased when exposed to hemoglobin at 25, 50 and 100 µM (see Supplementary Fig. S1d). Furthermore, the cooperative effects of hydrostatic pressure and hemoglobin on neurons viability were also studied *in vitro*. Compared with the exclusive treatment, hydrostatic pressure of 40 kPa with 25 µM of hemoglobin could decrease the neurons viability (Fig. 1a) and increase LDH releasing (Fig. 1b) significantly.

Different volumes (0, 25, 50 and 100 μL) of agarose gel were injected into the right striatum of rats to simulate the mass effect of hematoma after ICH *in vivo*. The moderate (50 µL) and severe (100 µL) mass effect from agarose gel led to LDH releasing significantly compared with the mild mass effect (25 µL) and Sham (Figs 1c, S1g). While, Gel (50 µL) + Hb (10 µL) and Gel (100 µL) + Hb (10 µL) also increased the LDH releasing significantly compared with the exclusive hemoglobin group.

These results indicated that the hydrostatic pressure had effect on neurons viability *in vitro* and neural activity *in vivo*. Furthermore, the hydrostatic pressure and hemoglobin, corresponding to the mass effect and followed biochemical effects in ICH, insulted the neural system together.

### Cellular Morphology and Tissue Architecture

The integrity of cellular morphology and tissue architecture is essential to the neural activity and function, but the hydrostatic pressure and hemoglobin trended toward lower expression levels of neuronal nuclear antigen (NeuN), microtubule-associated protein-2 (MAP-2), neural cell adhesion molecule L1 (NCAM-L1) and growth-associated protein 43 (GAP-43) *in vitro* (see Supplementary Fig. S2). Meanwhile, injection of the agarose gel or hemoglobin into the right striatum of rats also decreased the genes expression *in vivo* (see Supplementary Fig. S3), and the cooperative effects of hydrostatic pressure and hemoglobin disturbed their expression highly significantly.

**Figure 2:**
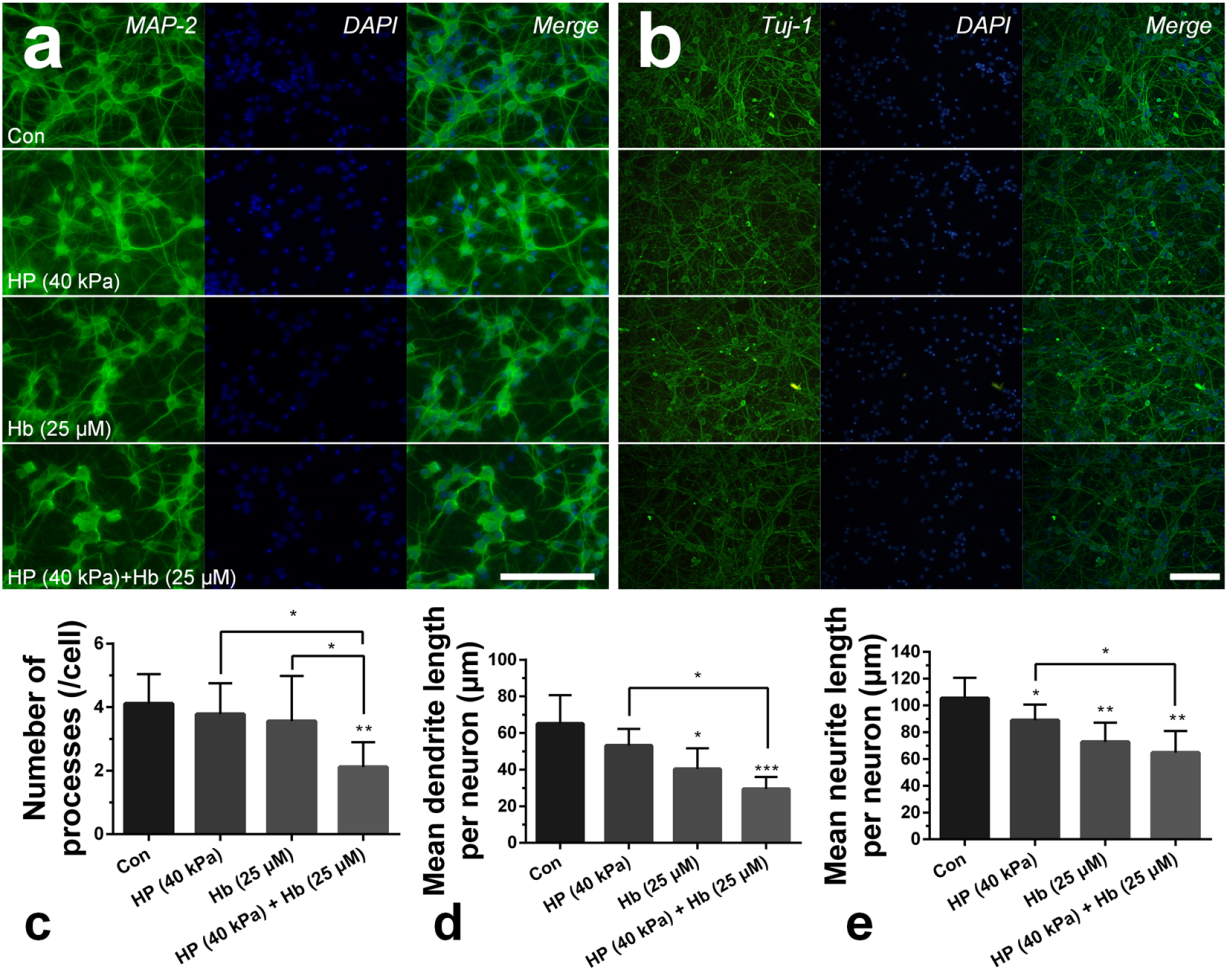
The elevated hydrostatic pressure or hemoglobin caused microtubule disruption (**a**) and structural degradation (**b**) *in vitro* (Scale bar: 100 μm). The number of processes (**c**) and mean dendrite length (**d**) were measured from MAP-2 staining. Neurite length (**e**) were measured from Tuj-1 staining. (**c**–**e**) were analyzed by Image-Pro Plus. Data are expressed as the means ± SD (n = 12, *P < 0.05, **P < 0.01, ***P < 0.001).

**Figure 3:**
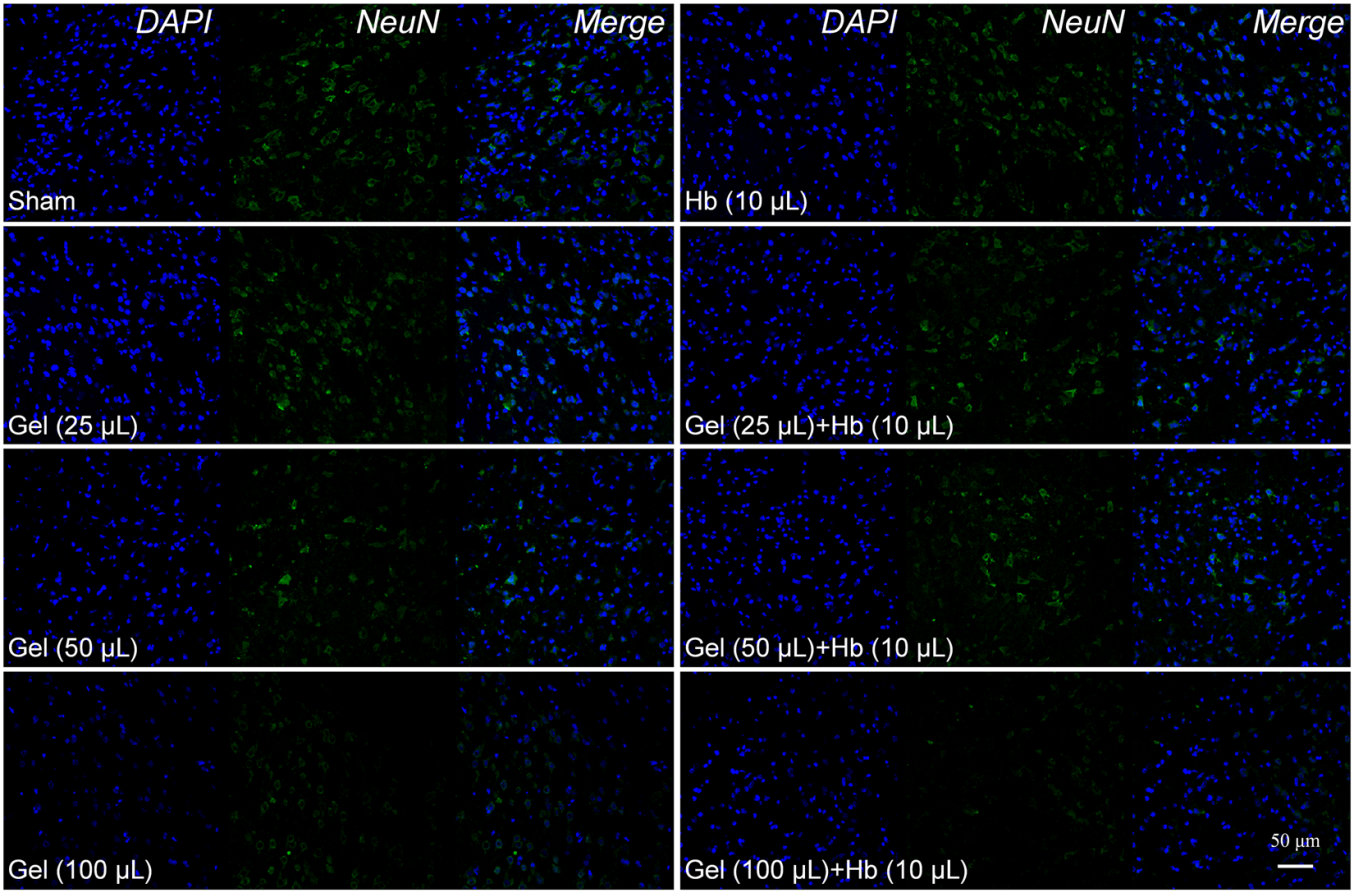
The ICH model decreased the expression of neurons markers (NeuN) *in vivo*.

**Figure 4:**
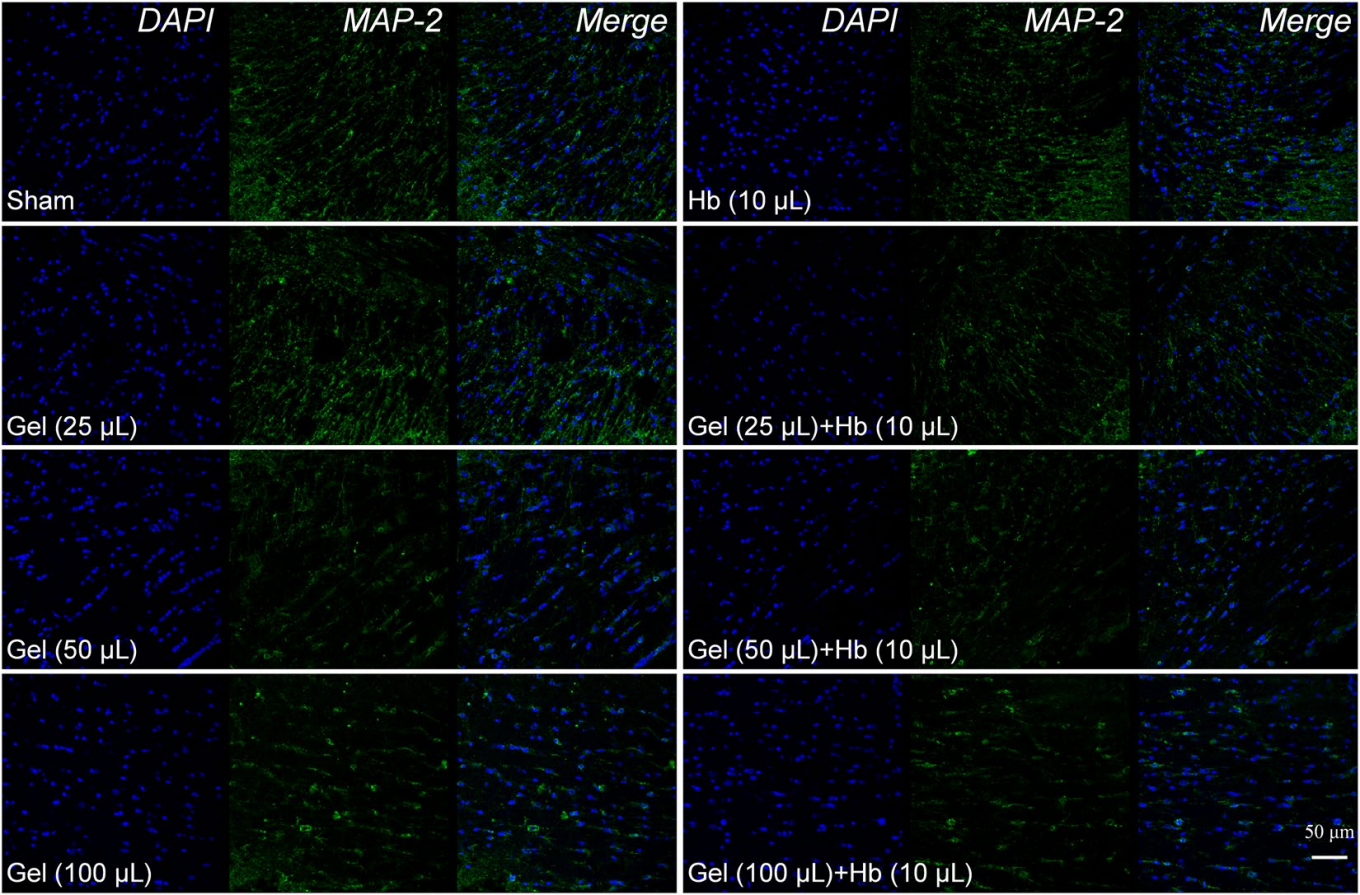
The ICH model decreased the expression of MAP-2 *in vivo*.

**Figure 5:**
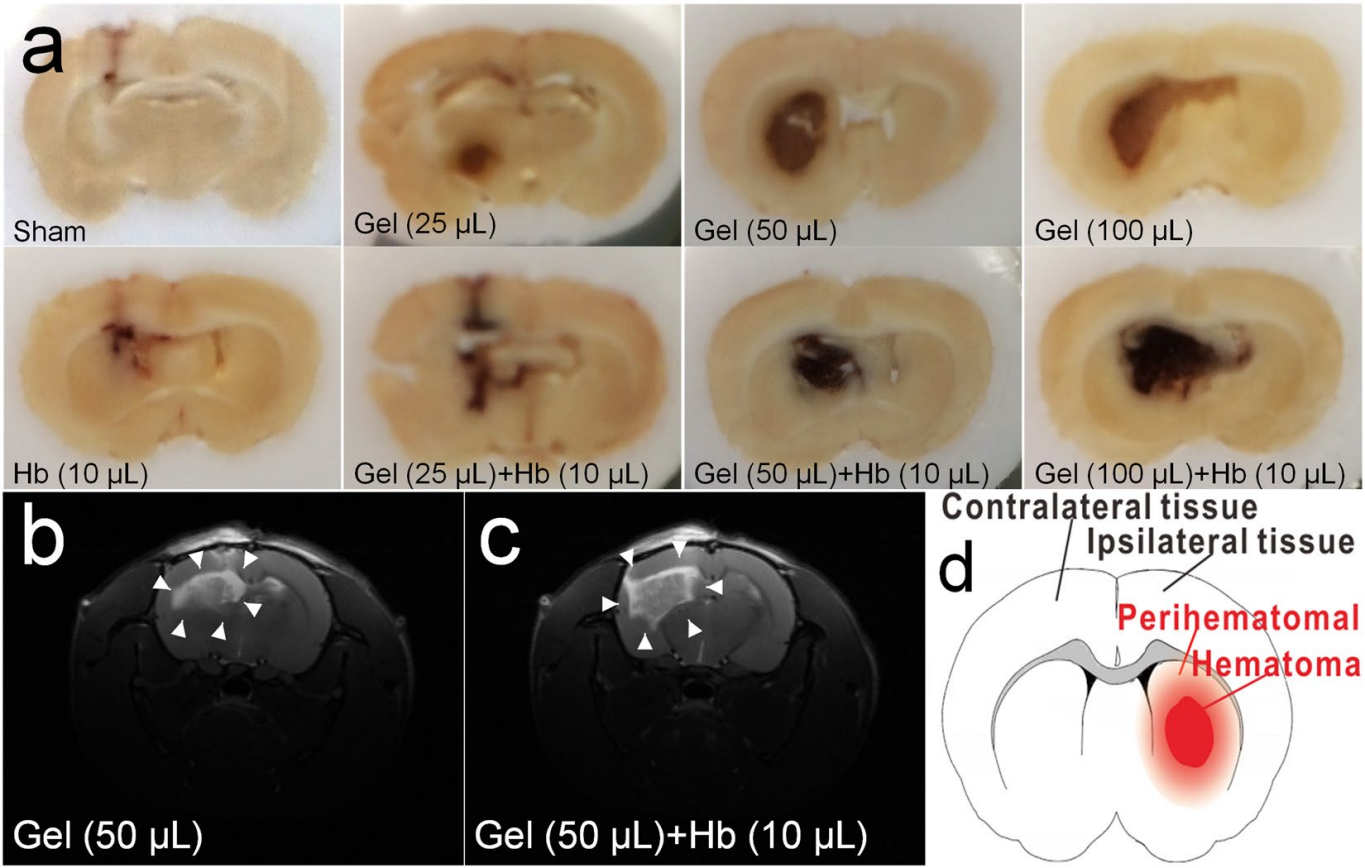
The images of coronal plane and MRI following ICH model after 24 h. (**a**) The images of coronal plane showed the insults from the agarose gel and hemoglobin on neural tissues. (**b**,**c**) Lesions were observed on MRI imaging (white arrows). (**d**) The hematoma and perihematomal tissues following ICH.

Axons and dendrites of neurons were stained by immunofluorescence, and the hydrostatic pressure and hemoglobin decreased the number and adhesion of neurons (Fig. 2), which also impeded and disrupted the dendrites and neurites independently and dependently. Furthermore, neurons were structural integrity but with the less, messy or broken processes which prevented the connection among neurons and decreased neurons adhesion (Fig. 2c–e). In addition, the proteins expression of NeuN and MAP-2 were also decreased *in vivo* which indicated less neurons and broken processes in the perihematomal tissues following ICH (Figs 3 and 4). Besides, images of coronal plane showed the insults from the agarose gel and hemoglobin on neural tissues which disordered the tissue architecture (Fig. 5a). The obvious mass effect from 50 µL agarose gel was observed on MRI imaging (Fig. 5b). Concurrent with the hemoglobin (10 µL), the mass effect induced significant edema in the perihematomal tissues (Fig. 5c).

### Neural apoptosis

**Figure 6:**
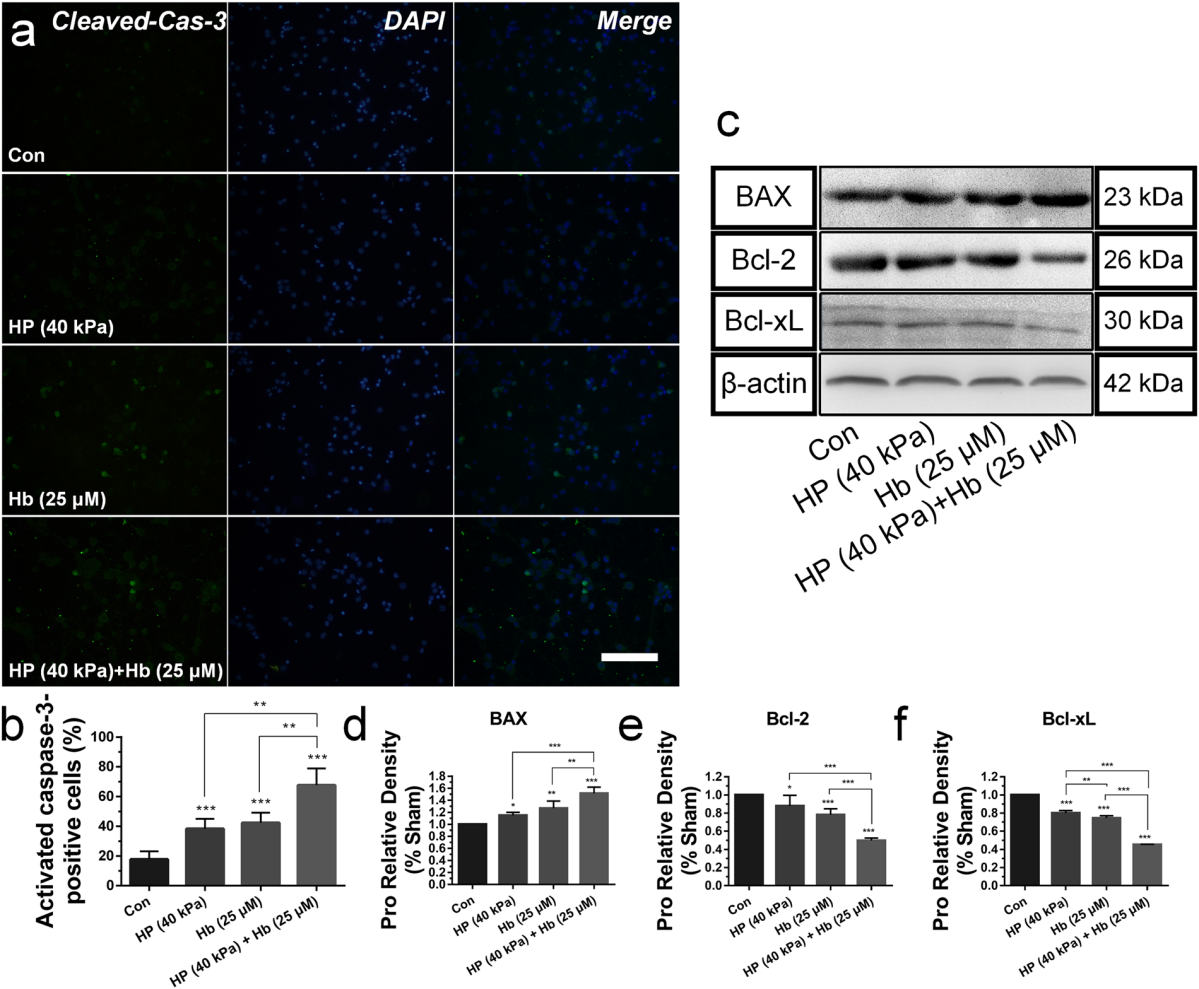
Effects of hydrostatic pressure and hemoglobin on neural apoptosis *in vitro*. (**a**) Representative fluorescence images immunostained with cleaved-caspase-3 in green and DAPI in blue (Scale bar: 100 μm). (**b**) Quantification of cleaved-Caspase-3-positive neurons, expressed as total DAPI-positive cells percentage. (**c**–**f**) The expression of proapoptotic protein of the BAX, Bcl-2 and Bcl-xL in neuronal samples after treated with hydrostatic pressure, hemoglobin or the mixture *in vitro*. The regions of interested proteins on the membranes were separated and proceeded as described in “Material and Methods” section. Data are expressed as the means ± SD (*n* = 12, *P < 0.05, **P < 0.01, ***P < 0.001).

The above results indicated that the hydrostatic pressure or cooperated with hemoglobin induced the decreased neurons viability *in vitro* and neural activity *in vivo*. We attempted to address these with neural apoptosis. Cleaved caspase-3, an activated form of caspase-3, indicated the increased apoptosis of neural cells^27^. The hydrostatic pressure increased the expression of cleaved caspase-3 *in vitro* compared to control samples (Fig. 6a,b), and the double staining also showed that hydrostatic pressure with hemoglobin significantly increased apoptosis and necrosis compared with the exclusive hydrostatic pressure or hemoglobin treatments (see Supplementary Fig. S4). Furthermore, the expression of proapoptotic protein of the Bcl-2 family (BAX), and antiapoptotic proteins (Bcl-2 and Bcl-xL) were detected to monitor the mitochondrial pathway of neural programmed cell death. The hydrostatic pressure highly increased the BAX expression, dramatically decreased the expression of Bcl-2 and Bcl-xL (Fig. 6c–f). While, the cooperative treatments significantly increased the BAX expression and decreased the Bcl-2 and Bcl-xL expression compared to the individual treatment.

**Figure 7:**
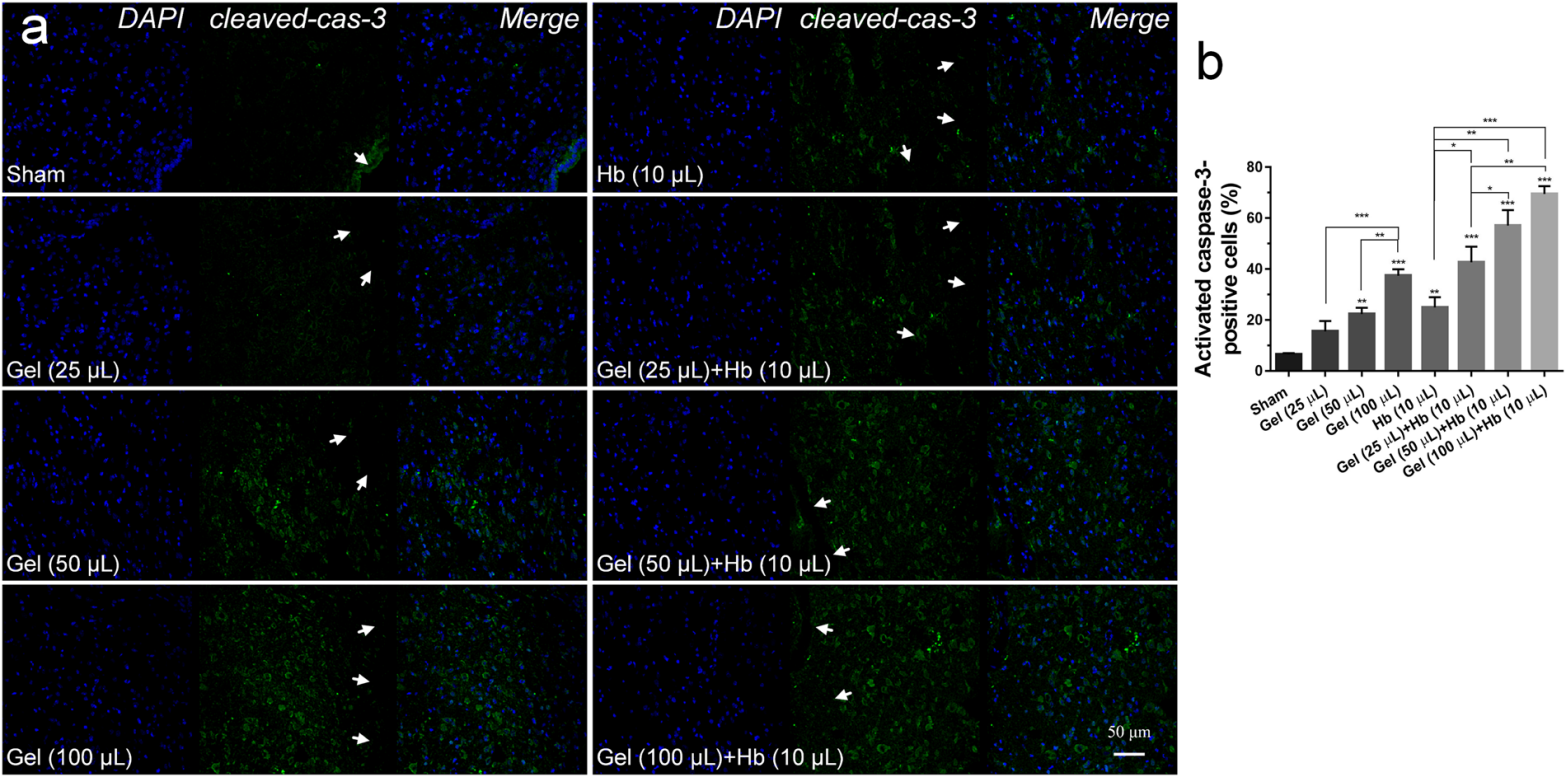
(**a**) Cleaved-caspase-3 immunoreactivity in the perihematomal tissues following ICH (Scale bar: 50 μm). The white arrow refered to the trace of needle which inserted stereotactically into the right striatum. (**b**) Quantification of cleaved-Caspase-3-positive neural tissues, expressed as total DAPI-positive cells percentage. Data are expressed as the means ± SD (*n* = 6, *P < 0.05, **P < 0.01, ***P < 0.001).

**Figure 8:**
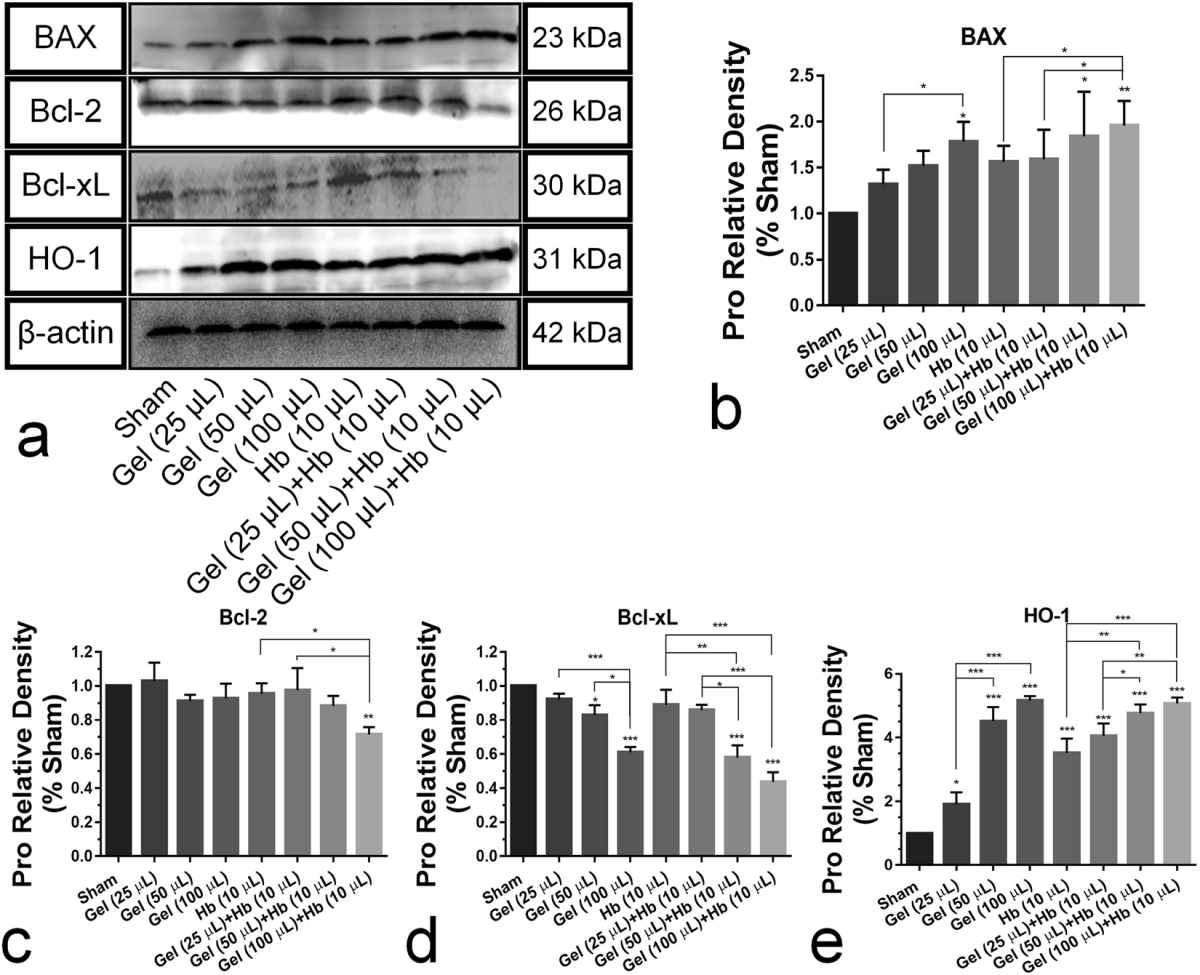
(**a**) Effects of hydrostatic pressure and hemoglobin on the expression of BAX, Bcl-2, Bcl-xL and HO-1 *in vivo*. (**b**–**e**) Refered to the expression of BAX, Bcl-2, Bcl-xL and HO-1, respectively. The regions of interested proteins on the membranes were separated and proceeded as described in “Material and Methods” section. Data are expressed as the means ± SD (*n* = 12, *P < 0.05, **P < 0.01, ***P < 0.001).

Moreover, brain injection of the agarose gel and hemoglobin also induced higher protein level of cleaved caspase-3 after ICH *in vivo* compared with sham group (Fig. 7), and the expanded gel worsened the neural apoptosis (Fig. 8a–d). Heme oxygenase-1 (HO-1), a key enzyme of heme degradation, was also elevated and accelerated heme degradation and iron deposition (Fig. 8a,e), which resulted in the secondary injuries following the hydrostatic pressure.

**Figure 9:**
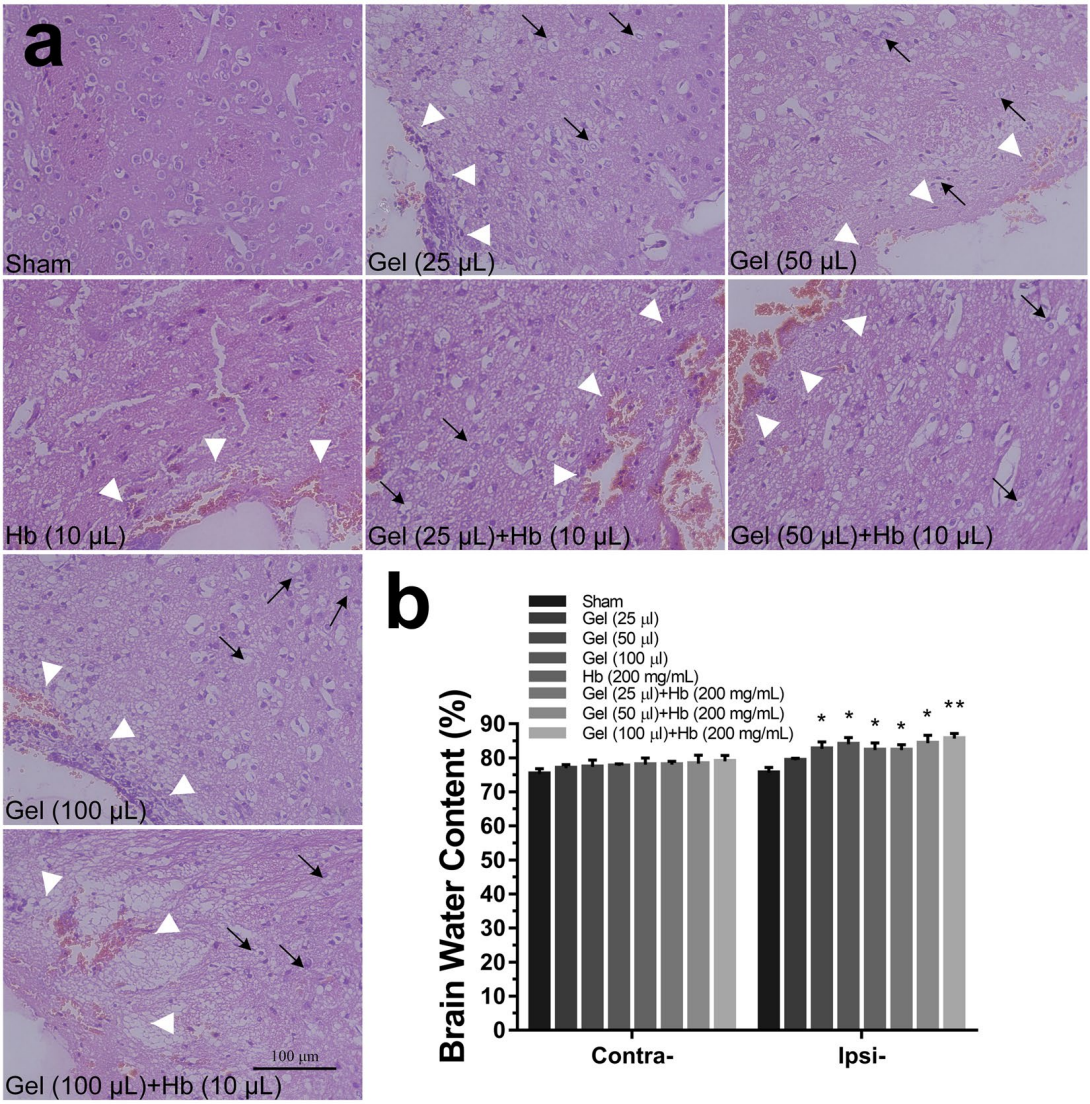
H&E staining (**a**) and brain water content (**b**) at 24 h after treated with hydrostatic pressure, hemoglobin or the mixture *in vivo*. The black arrow refered to the cell necrosis (nuclear pyknosis, karyorrhexis and karyolysis) *in vivo*. The white arrow refered to the trace of needle which inserted stereotactically into the right striatum. (Scale bar: 100 μm). Data are expressed as the means ± SD (*n* = 6, *P < 0.05, **P < 0.01, ***P < 0.001).

H&E staining showed that neural cells in the perihematomal tissues following ICH were morphologically normal in Sham. While, cell necrosis (nuclear pyknosis, karyorrhexis and karyolysis) can be observed after injecting gel and hemoglobin, and treatment by gel plus hemoglobin could significantly accelerate the cell necrosis (Fig. 9a). Moreover, an increase in hydrostatic pressure conducted by brain injection of gel increased the perihematomal water content, but no significant difference in brain edema was showed between the cooperative treatment and exclusive hydrostatic pressure (Fig. 9b).

### Piezos expression

**Figure 10:**
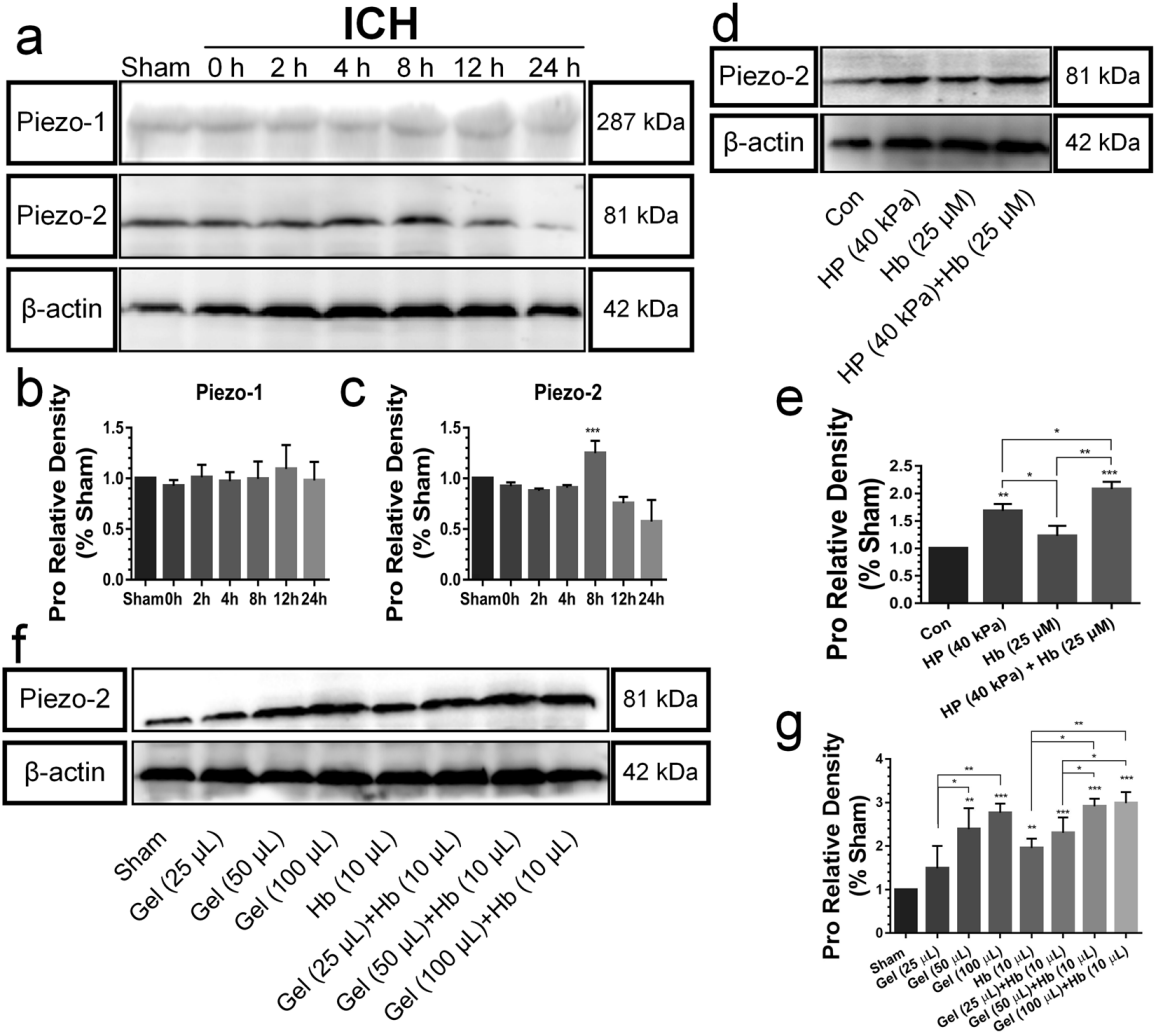
(**a**–**c**) The neural expression of Piezo-1, 2 following ICH at different time. (**d**,**e**) The expression of Piezo-2 after exposing to the hydrostatic pressure (40 kPa) with or without hemoglobin for 8 h *in vitro*. (**f**,**g**) The expression of Piezo-2 after injection of gel or the mixture of gel and hemoglobin into the right striatum of rats for 8 h *in vivo*. The regions of interested proteins on the membranes were separated and proceeded as described in “Material and Methods” section. Data are expressed as the means ± SD (*n* = 12, *P < 0.05, **P < 0.01, ***P < 0.001).

It has been increasingly demonstrated that Piezos-mediated mechanotransduction was important in mechanically activated channels^28^. In this paper, Piezo-1 had no obvious change compared with sham group. However, the protein level of Piezo-2 reached a plateau at 8 h after ICH *in vivo* (Fig. 10a–c), so the remaining experiments adopted this time point. A significantly higher Piezo-2 expression was caused by the exclusive hydrostatic pressure (40 kPa) *in vitro* (Fig. 10d,e). Though the hemoglobin had no significant effect, the cooperative effects increased its expression highly. Besides, the significant increase in Piezo-2 expression can be observed by brain injection of gel (50 and 100 µL) *in vivo* compared to the sham group (Fig. 10f,g). In addition, the Piezo-2 expression was increased highly following the expanded volume of gel in the Gel groups or Gel + Hb groups. The increased expression of Piezo-2 in Gel + Hb groups might also be due to the growing mass effect of additional edema which caused by hemoglobin or other biochemical pathways (Fig. 5c), which is also accounted for the reason why hemoglobin upregulated Piezo-2 expression *in vivo* rather than *in vitro*. While, the exogenous stimulus was exclusive and artificially controlled *in vitro*. This suggested that Piezo-2 had a prominent role in sensing hydrostatic pressure following ICH.

## Discussion

The primary and secondary injuries after ICH can result in the severe neurological deficits or even death^7^. Hematoma generated after the onset of ICH, and mass effect was induced as hematoma expansion which would exist during the pathogenetic process. Therefore, the present study focused on the damage mechanism of hydrostatic pressure associated with mass effect and the cooperative effect of hydrostatic pressure plus hemoglobin on neural injuries. We have performed a series of experiments to verify the effects of hydrostatic pressure and hemoglobin independently and cooperatively.

A myriad of studies has demonstrated that hydrostatic pressure could be simulated by the custom-designed device *in vitro*, such as the water column, hydraulic cylinder, and pressurized or pumped gas. For instance, the intercellular calcium signaling was disturbed in 276 kPa just for 20 msec.^15^. Dorsal root ganglion neurons exposing to 0.1–0.7 MPa for 10 min would generate apoptosis^16^. The cultured retinal ganglion cells showed the increased apoptotic rate and ROS production under 50 mm Hg for 6 h^29^. In the present study, we simulated the elevated hydrostatic pressure following ICH by exposing cortical neurons to 20–40 kPa pressurized gas (95% air and 5% CO_2_) for 24 h.

In addition, the ICH animal models have been established by injecting autologous arterial blood or collagenase into the right striatum of animals^30,31^, which were usually used to investigate the ICH-induced injuries. However, the traditional ICH animal models can’t be used to investigate the exclusive hydrostatic pressure following ICH because of the products of coagulation and hemoglobin breakdown. Implanting the microballoon^17^ or loading the water column on cerebral cortex^32^ have provided the tools for exploring hydrostatic pressure-induced injuries after ICH, but they were fairly time consuming, complex operation, and totally different from the common ICH. Here, the agarose gel (25–100 µL) were injected into the right striatum of rats to study the effect of exclusive hydrostatic pressure on injuries after ICH. As shown in Figs 5a and S5, few iron depositions can be observed after injection of agarose gel, which successfully avoided the influence of hemoglobin. Besides, the MRI imaging also showed the obvious mass effect form hematoma and edema with a sharp boundary (Fig. 5b,c). 50 µL agarose gel (Fig. 5b) induced severe mass effect but didn’t cause significant edema in the perihematomal tissue compared with the cooperative group (Fig. 5c). Overall, the *in vitro* and *in vivo* models in this paper were easy to operate to investigate the exclusive hydrostatic pressure on neural injuries.

The mentioned hydrostatic pressure gives rise to two mechanical effects: the transverse stress through the cross section of the hematoma, and the tensile stress which acts on the surrounding tissues^11,33^. We found that the hydrostatic pressure suppressed neural viability independently in this study, and microtubule disorganization and structural degradation were also observed. Furthermore, the hydrostatic pressure triggered downstream events such as apoptotic pathways^7^. Here, the hydrostatic pressure associate with ICH could upregulate the expression caspase-3 and BAX, and downregulate the expression Bcl-2 and Bcl-xL.

Besides the independent effects, hydrostatic pressure and hemoglobin corresponding to the mechanical stimuli and the followed biochemical effects insulted neural tissues in a cooperative manner following ICH. Results indicated that the hydrostatic pressure enhanced the toxicity of hemoglobin, and larger volume of gel induced bigger mass effect could result in more iron deposit. Therefore, the hydrostatic pressure not only gave rise to the neurological injuries, but also exacerbated the subsequent injuries from hemoglobin or other biochemical pathways further^11,34^. To date, numerous researches focused on the products from blood, but neglected the mechanical and their cooperative effects.

Moreover, the hydrostatic pressure insulted neural tissue following the *in vitro* and *in vivo* ICH models in this study, and the mechanism of mechanotransduction was also investigated. We speculated that Piezos might play important roles in the onset and development of ICH. Results indicated that the exclusive hydrostatic pressure could upregulate the Piezo-2 expression significantly, which reached a plateau at 8 h after ICH. Furthermore, hemoglobin increased Piezo-2 expression significantly *in vivo*, and the elevated volume of Gel also promoted its expression significantly in the cooperative groups. Therefore, Piezo-2-mediated mechanotransduction had a key role in brain injuries after ICH.

The present study had several limitations. Now, the basic research and clinical management only focused on the biochemical effects, and the cellular and molecular effects induced by the increased IP following ICH was absent, which resulted in inadequate experimental evidences. However, in this paper, we provided the preliminary insight into the relationship between the neural injuries and the increased hydrostatic pressure which had been investigated in glaucoma^35^ and cartilage repair^36^. Meanwhile, we also took the Piezo-2-mediated mechanotransduction into consideration in ICH. This finding disclosed that the mechanical stimuli induced neural injuries following ICH and the preliminary related mechanism.

In summary, we have successfully constructed the *in vitro* and *in vivo* ICH models to investigate the damage mechanism of hydrostatic pressure associated with mass effect after ICH and the cooperative effect of hydrostatic pressure plus hemoglobin on neural injuries. Results indicated that the hydrostatic pressure had significant effect on neural viability, structure and apoptosis, which also contributed to the subsequence secondary injuries. Furthermore, Piezo-2-mediated mechanotransduction had a key role in brain injuries following ICH. In addition, the toxicity of hemoglobin would be enhanced when conducted with hydrostatic pressure together. Therefore, hydrostatic pressure induced by mass effect not only gave rise to brain injuries directly, but also increased the toxicity of hemoglobin in the progress of secondary brain injury after ICH.

## Methods

### Primary Cortical Neuronal Cultures

Primary cortical neurons were prepared from D18–19 embryos carried by Sprague-Dawley dam according to the previous method^31^. All procedures were in accordance with the institutional guidelines for animal experimentation and approved by the Institutional Animal Care and Use Committee of Third Military Medical University, China. Briefly, cortices were removed, triturated and digested, and the cell suspension was filtrated and centrifuged. The cell pellets were extracted and resuspended. After the first 4 h, the culturing medium was replaced by neurobasal media (Gibco, Rockville, MD, USA) with 2% B27 (Gibco), 0.5 mM GlutaMAX (Gibco) and 0.5% penicillin-streptomycin (Gibco). Neurons were cultured for 7 days for *in vitro* experiments.

### Animal Preparation and Intracerebral Gel Injection

The *in vivo* ICH model was adapted from an established rat protocol^4^. The male Sprague–Dawley rats weighing 250 to 300 g were used in this study. Needle was inserted stereotactically into the right striatum (coordinates: 0.2 mm anterior, 5.5 mm ventral, 4.0 mm lateral to the bregma), and agarose gel (1%, w/v) was injected to simulate the mass effect of hematoma after ICH *in vivo*. Sham had only intracerebral needle insertion.

### Experimental Groups

For *in vitro* model: the custom-designed device was consisted of a pressure vessel, a pressure meter, a regulator, pressurized gas and high-pressure connecting tubing. Cell culture plates were placed into the pressure vessel and exposed to the cell culture incubator. The pressurized gas (95% air and 5% CO_2_) was controlled by the regulator. The pressure rose from 0 to 60 kPa in about 10 min, and pH of the culturing medium had no significant changes in 24 h (see Supplementary Fig. S6). Neurons were exposed to hydrostatic pressure (HP: 0, 20, 30, 40 kPa for 24 h), hemoglobin (Hb: 0, 6.25, 12.5, 25, 50, 100 µM for 24 h. Hemoglobin was dissolved in 0.2% dimethyl sulfoxide.) (Sigma, St. Louis, MO, USA), or HP (40 kPa) with Hb (25 µM) for 24 h.

For *in vivo* model: Eight groups of six rats each were used for MRI, RT-qPCR and Western Blotting (six rats per group for these experiments). ICH rats were established by injecting agarose gel (0, 25, 50, 100 µL) and followed with or without hemoglobin (3 mM, 10 µL) in the right striatum of rats.

ICH rats were established by injecting Gel (50 µL) to monitor the expression of Piezos at 0, 2, 4, 8, 12 and 24 h. Six rats in each group were used.

And injecting Gel (50 µL) or Gel (50 µL) with hemoglobin (3 mM, 10 µL) for 24 h in MRI experiment.

### Cell Viability Assay

Neuron viability was determined by CCK-8 assay (Dojindo Laboratories, Kumamoto, Japan), according to the manufacturer’s instructions. The release of LDH was detected using the LDH assay kit (Jiancheng Bioengineering, Nanjing, China), and LDH would increase if the cellular injuries occurred^37^. In addition, the apoptosis and necrosis of neurons were stained by Hoechst 33342/PI detection kit (Beyotime Biotechnology, Shanghai, China).

### Brain water content and Histology

The brain water content and iron deposition were detected according to the previous study^38^. Eight groups of six rats each were used for each experiment. The brain tissue slice was divided into 2 hemispheres along the midline, and brain samples were immediately weighted to obtain the wet weight, and dried at 100 °C for 24 h to obtain the dry weight. The formula was the following: (wet-dry)/wet weight. Iron deposition was evaluated by Perls’ staining. The area was quantified by ImageJ^39^. Besides, the ICH rats were sacrificed at 24 h, and the cerebrum was fixed with 4% paraformaldehyde, embedded by paraffin, and stained with Hematoxylin-Eosin.

### RNA Preparation and RT-qPCR

Total RNA was isolated by Trizol reagent (TaKaRa Biotechnology, Dalian, China). RT-qPCR was performed with SYBR Green (TaKaRa Biotechnology). The primer sequences showed in Supplementary Table S1.

### Magnetic resonance imaging

MRI was performed on a BRUKER BioSpec70/20USR after 24 h following ICH surgery. Rats were anesthetized by isoflurane and placed in a stereotaxic head holder within the magnet. T_2_weighted imaging was acquired using a multislice (22 slices) and multiecho (6 echoes) sequence, with repetition time/echo time = 3000/30 ms. Images were produced using a 50 × 55 mm^2^ field of view, 1 mm slice thickness and 250 × 250 matrix.

### Western Blotting

Protein was extracted, and concentrations were detected by BCA assay kit (Beyotime). To separate the regions where the target protein appeared, membranes were cut along the molecular weight marker. Incubating with rabbit-anti-Bax, rabbit-anti-Bcl-2, rabbit-anti-Bcl-xL, rabbit-anti-HO-1 (Santa Cruz, Dallas, TX, USA), Piezo-1, 2 (Abcam, Cambridge, MA, USA) and β-actin (Beyotime) antibodies, followed by the secondary antibody (Beyotime). Bands were visualized by ECL and intensities were quantified by Quantity-one. Some representative strips were proceeded with “Microsoft Office PowerPoint 2016”.

### Immunofluorescence

Eight groups of six rats each were used. Samples were incubated with rabbit-anti-MAP-2, rabbit-anti-NeuN, rabbit-anti-neuron-specific tubulin-1 (Tuj-1) (Santa Cruz), and rabbit-anti-cleaved caspase-3 (Cell Signaling Technology, Beverly, MA, USA), followed with Alexa Fluor 488-Labeled Goat Anti-Rabbit IgG (Beyotime). All samples were counterstained with DAPI. Representative fluorescence images were photographed under a fluorescent microscope (Olympus) or confocal microscope (Leica), and quantified by ImageJ^39^. The number of processes, length of dendrite and neurite were analyzed and normalized by the neuron number. Ten random fields were analyzed in each independent experiment, which was replicated for 6 times.

### Statistics

All data were presented as means ± S.D. A paired-samples T test was used for two groups and one-way ANOVA for more than two groups were performed using SPSS software, version 23.0. Difference with a value of *P* < 0.05 was considered statistically significant.

## Electronic supplementary material

Supplementary Information
