# Supplementary material for: Neural Injuries Induced by Hydrostatic Pressure Associated With Mass Effect after Intracerebral Hemorrhage
Source: Sci Rep. 2018 Jun 15;8:9195. doi: 10.1038/s41598-018-27275-7 (PMC6003942; doi:10.1038/s41598-018-27275-7)
Supplement: Supplementary file 1 — Supplementary Information [file 41598_2018_27275_MOESM1_ESM.docx]

Neural Injuries Induced by Hydrostatic Pressure Associated With Mass Effect after Intracerebral Hemorrhage

Tingwang Guo, PhD^1,2^, Peng Ren, PhD^1,2^, Xiaofei Li, MA^1,2^, Tiantian Luo, MA^1,2^, Yuhua Gong, PhD^1,2^, Shilei Hao, PhD^1,2,*^, Bochu Wang, PhD^1,2,*^

^1^ Key Laboratory of Biorheological Science and Technology, Ministry of Education, College of Bioengineering, Chongqing University, Chongqing 400030, China

^2^ Collaborative Innovation Center for Brain Science, Chongqing University, Chongqing 400030, China.

ONLINE SUPPLEMENT

**Supplemental Tables**

| Gene | Forward primer | Reverse primer |
| --- | --- | --- |
| NeuN (neuronal nuclear antigen) | ACGGAGCGGCACTGGAGC | ACGACCCTGGAAGCAAACGG |
| MAP-2 (microtubule-associated protein-2) | CAAAGTTTATGGGGAGAAAAGGGA | CAAGGGCAATAGAATCAAGGCAAG |
| NCAM-L1 (neural cell adhesion molecule L1) | CACCAGTGAGAGGGTGAGTG | CTCCAGTACATGGTGTCCTTT |
| GAP-43 (growth-associated protein 43) | AACGGAGACTGCAGAAAGCA | GCCTCGGGGTCTTCTTTACC |
| GAPDH | GATGGTGAAGGTCGGTGTGA | GGGATCTCGCTCCTGGAAG |

**Table S1**. Primer designs for PCR. NeuN, neuronal nuclear antigen; MAP-2, microtubule-associated protein-2; NCAM-L1, neural cell adhesion molecule L1; GAP-43, growth-associated protein 43.

**Supplemental Figures**


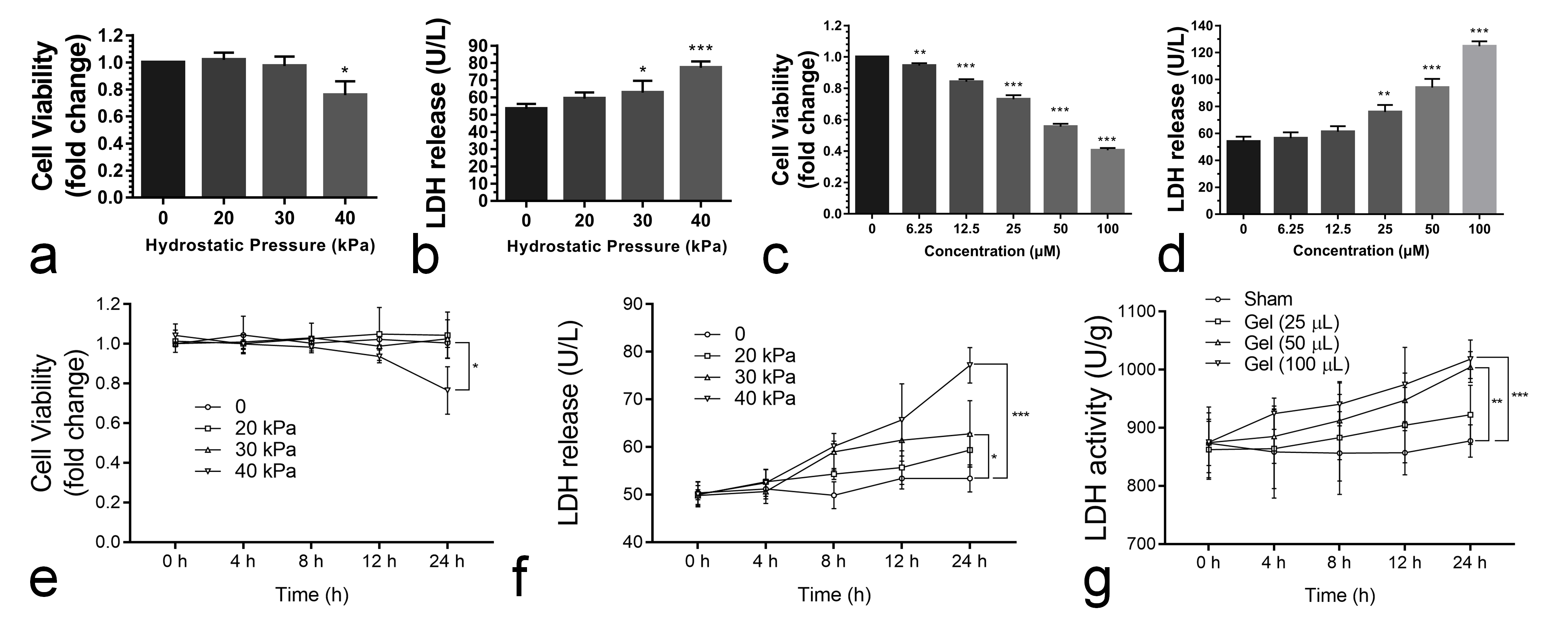


**Figure S1.** The cell viability and LDH release of neurons after exposing to hydrostatic pressure (a and b) or hemoglobin (c and d) for 24 h. The cell viability (e) and LDH release of neurons (f) or neural tissues (g) after establishing models for 4, 8, 12 and 24 h. Data are expressed as the means ± SD (*n* = 12, **P* < 0.05, ***P* < 0.01, ****P* < 0.001).


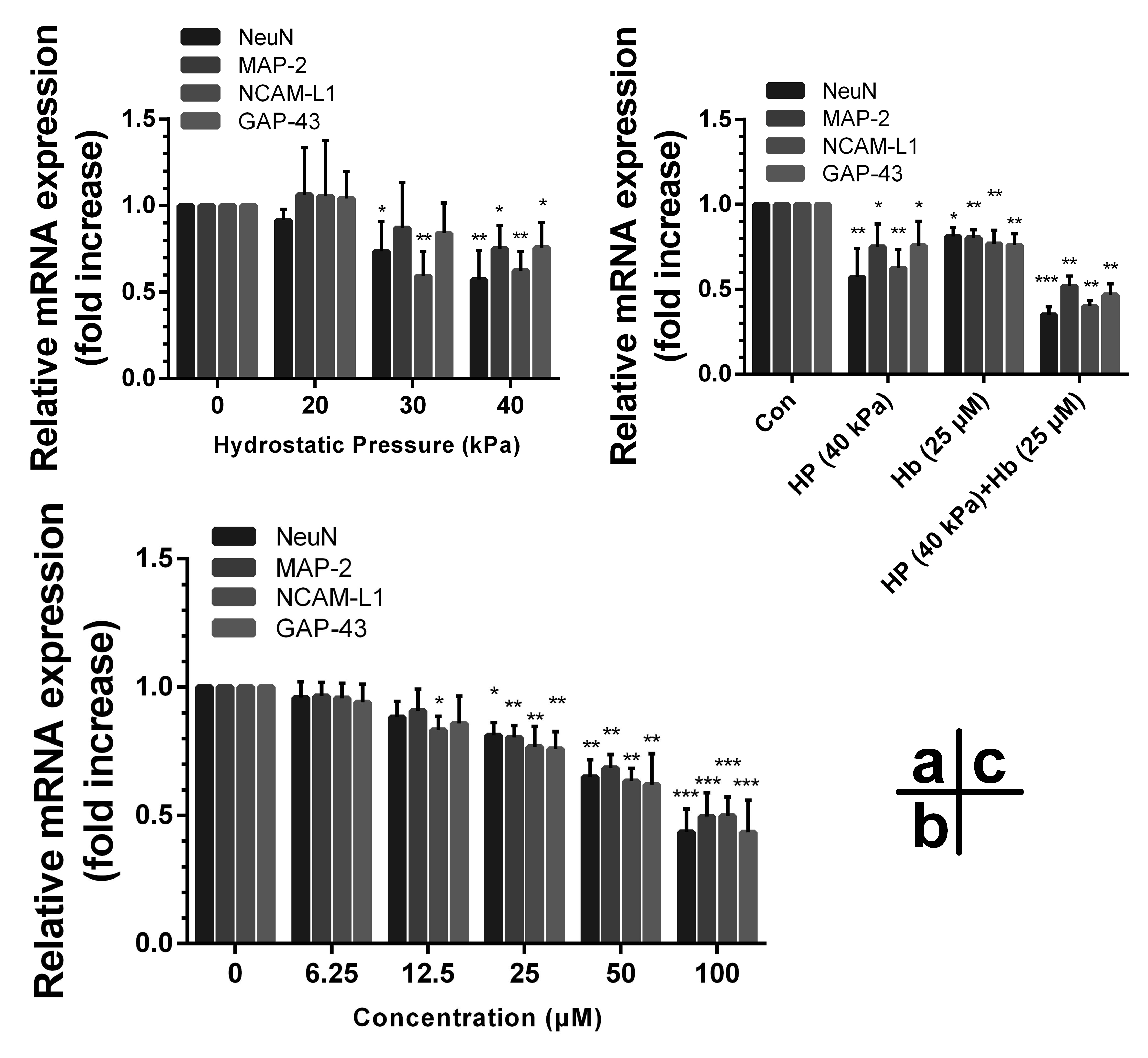


**Figure S2.** The disturbed expression of genes related to the neurons structure after exposing hydrostatic pressure (a) or hemoglobin (b) and the mixture (c) for 24 h *in vitro*. Data are expressed as the means ± SD (*n* = 12, **P* < 0.05, ***P* < 0.01, ****P* < 0.001).


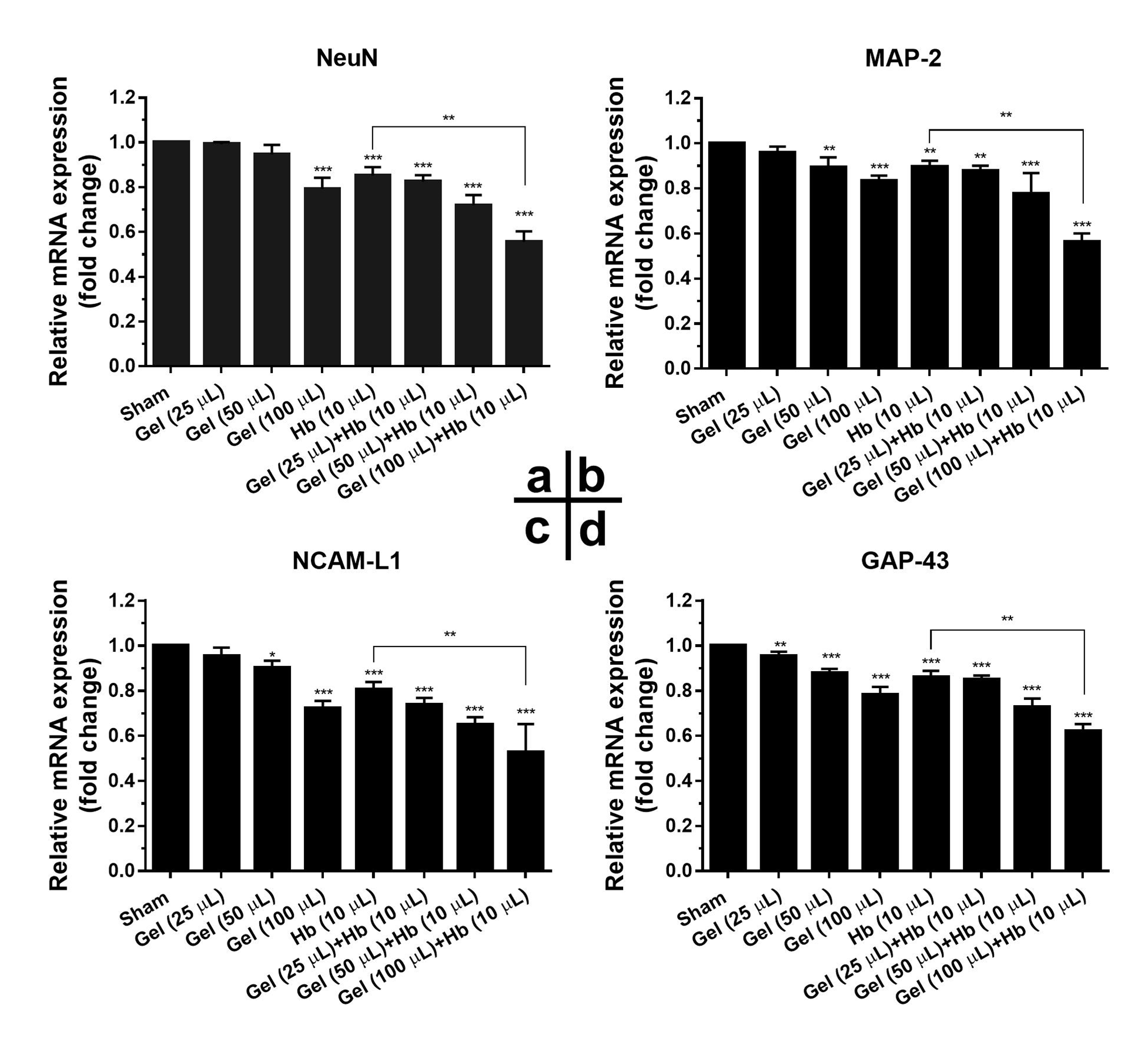


**Figure S3.** The disturbed expression of genes related to the neural structure after treated with hydrostatic pressure, hemoglobin or the mixture *in vivo*. Data are expressed as the means ± SD (*n* = 12, **P* < 0.05, ***P* < 0.01, ****P* < 0.001).


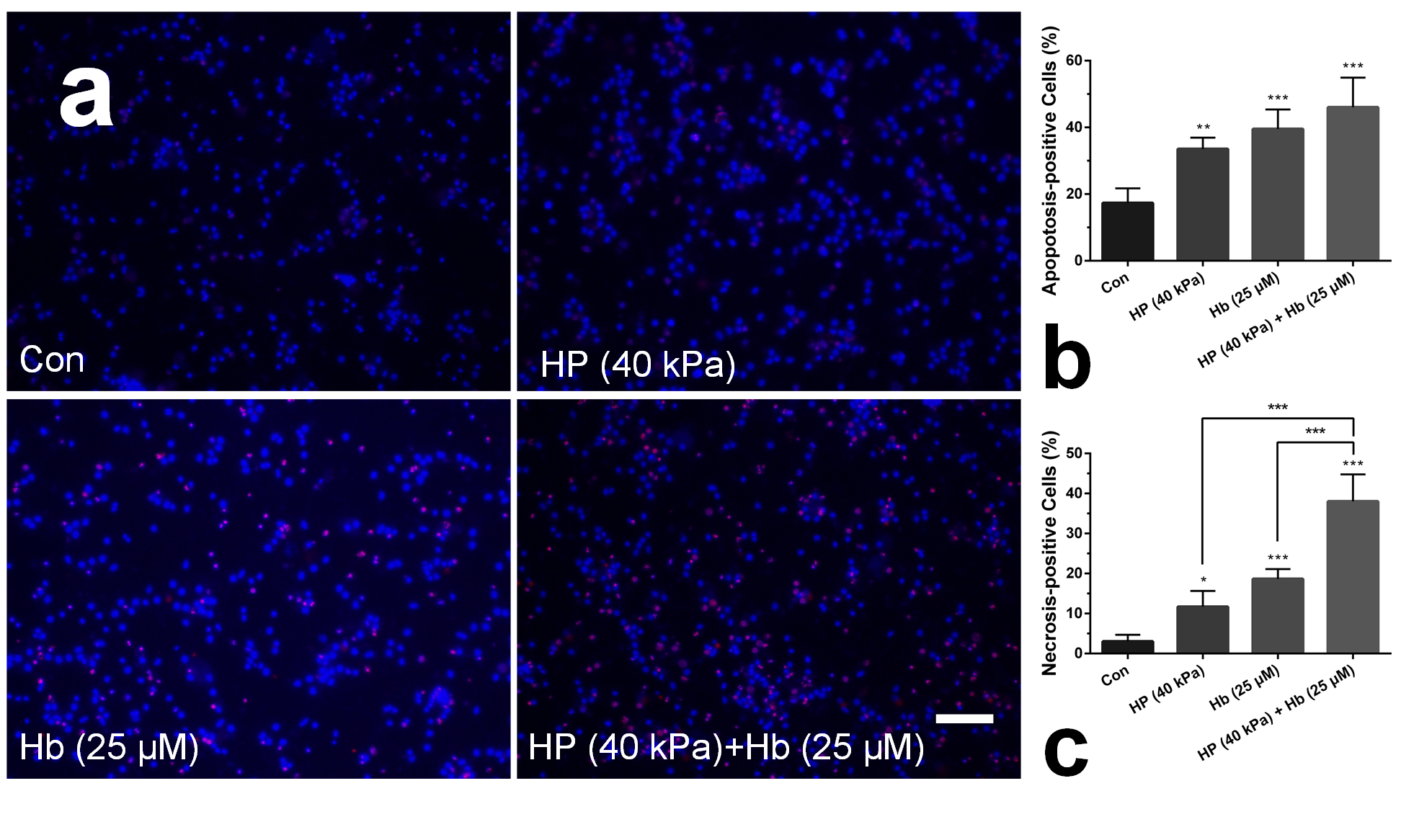


**Figure S4.** The hydrostatic pressure and hemoglobin induced neural apoptosis and necrosis. (a) Double staining by Hoechst 33342/PI showed that hydrostatic pressure (40 kPa) with/without hemoglobin (25 µM) induced significant apoptosis and necrosis (Scale bar: 100 μm). Quantification of apoptosis-positive (b) and necrosis-positive (c) neurons were measured from Hoechst 3342 (blue) and PI (red) staining, respectively. Data are expressed as the means ± SD (*n* = 12, **P* < 0.05, ***P* < 0.01, ****P* < 0.001).


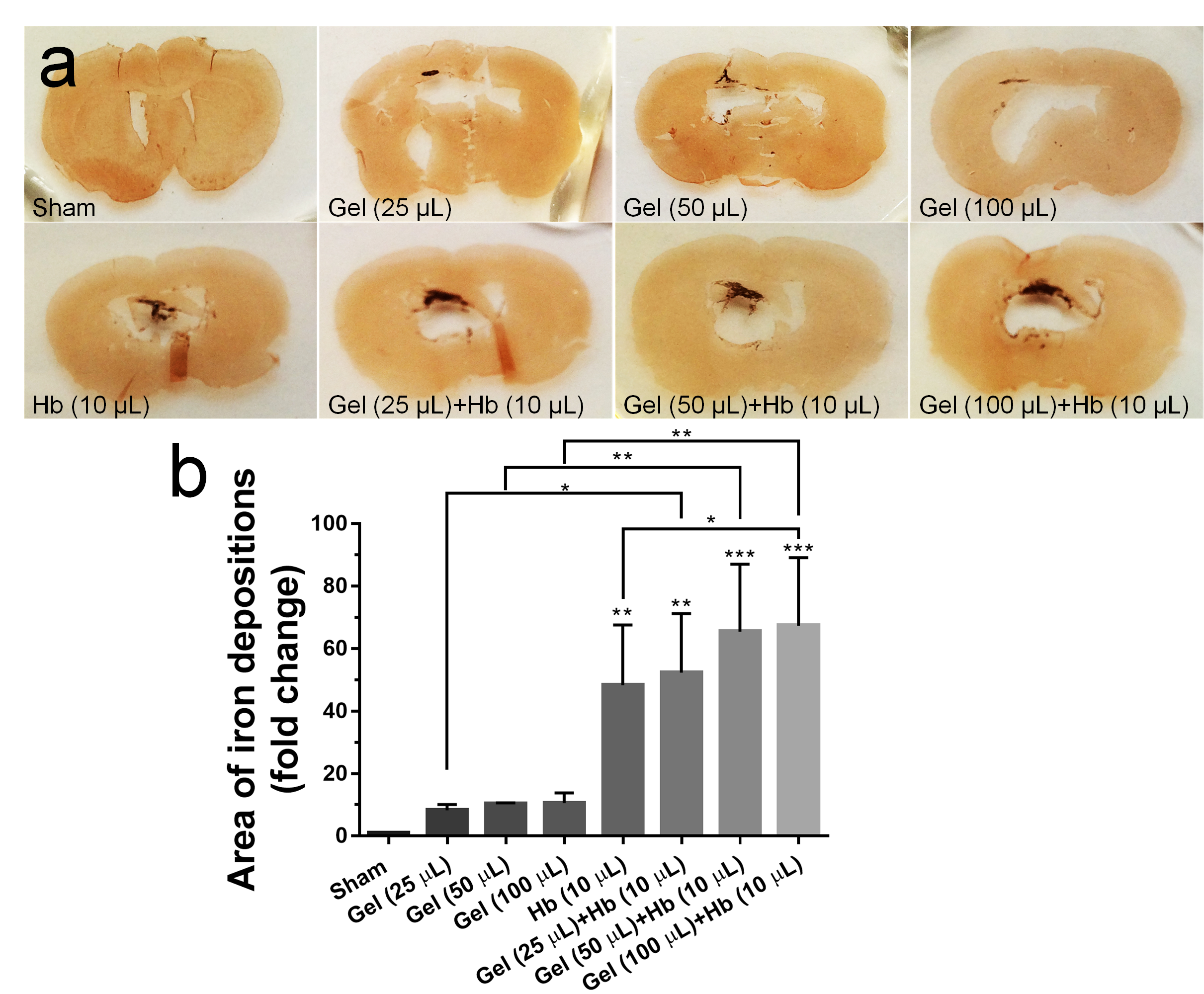


**Figure S5.** The iron depositions after 24 h (Perl’s staining). The representative images (a) and area (b) of iron depositions. Data are expressed as the means ± SD (*n* = 3, **P* < 0.05, ***P* < 0.01, ****P* < 0.001).


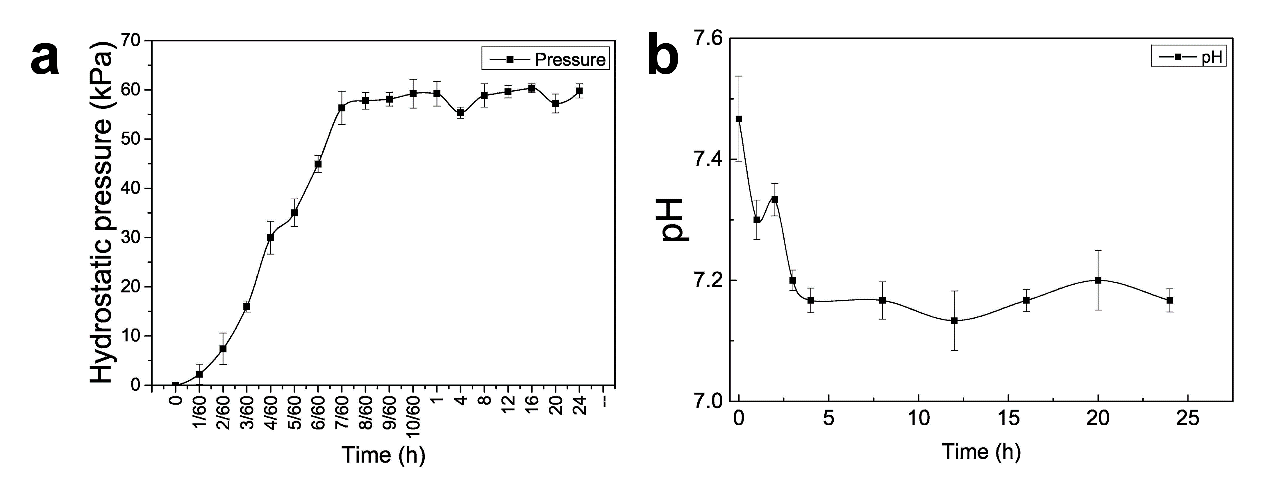


**Figure S6.** Loading the hydrostatic pressure on primary hippocampal neurons by the custom-designed device. (a) The hydrostatic pressure rose from 0 to 60 kPa in about 10 min and could keep in 60 kPa (± 2 kPa) last for 24 h. (b) No significant change in the pH of culturing medium for 24 h.
